# Supplementary material for: Evaluating the feasibility and acceptability of an exercise and behaviour change intervention in socioeconomically deprived patients with peripheral arterial disease: The textpad study protocol
Source: PLoS One. 2022 Jun 24;17(6):e0269999. doi: 10.1371/journal.pone.0269999 (PMC9231723; doi:10.1371/journal.pone.0269999)
Supplement: S1 File — (PDF) [file pone.0269999.s002.pdf]

RESEARCH PROTOCOL

V1.6

Evaluating the feasibility and acceptability of an exercise and behaviour change intervention in socioeconomically deprived patients with peripheral arterial disease:

THE TEXTPAD STUDY

Research Protocol Version 1.6
19/03/2021

Funded by League of Friends of Freeman Hospital and departmental funds
IRAS Reference Number: 286735
R&D Reference Number: 09623

CONTENTS

1. RESEARCH PERSONNEL CONTACTS 4

1.1. Principal Investigator 4

1.2. Co-investigators 4

2. PROTOCOL SIGNATURE PAGE 7

2.1. Principal investigator 7

2.2. Co-investigators 7

3. BRIEF PROTOCOL SUMMARY 8

4. BACKGROUND 9

5. PRIMARY RESEARCH QUESTION 10

6. AIMS and OBJECTIVES OF THE STUDY 10

6.1. Primary aim 10

6.2. Primary objectives 10

6.3. Secondary aims 10

6.4. Secondary objectives 10

6.5. Tertiary/exploratory aims and objectives 11

7. STUDY DESIGN 11

7.1. Participants 11

Inclusion criteria 11

Exclusion criteria 11

7.2. Research staff 12

7.3. Recruitment procedures 14

7.4. Consent 14

7.5. Randomisation 14

8. INTERVENTION 14

Smoking Cessation 15

Alcohol intervention 15

Nutrition 15

Mental well-being and finances 15

Supervised exercise 16

8.1. Usual care 16

9. OUTCOME MEASUREMENTS 18

9.1. Primary outcomes 18

9.2. Secondary outcomes 19

Objective functional capacity 19

Subjective functional capacity 19
Quality of life 20
Physical activity levels 20
Sleep 21
Alcohol and tobacco use 21
Mental wellbeing 21
Patient activation 21

10. STUDY POWER 22

11. DATA ANALYSIS 22

12. HEALTH ECONOMICS 23

13. STUDY MONITORING 23

14. SERIOUS ADVERSE EVENT MONITORING AND REPORTING 23

15. ETHICS AND REGULATORY ISSUES 23

16. PATIENT AND PUBLIC INVOLVEMENT 24

17. CONFIDENTIALITY & DATA HANDLING 24

18. INFORMATION GOVERNANCE/DIGITAL PLATFORM 24

19. INSURANCE AND FINANCE 25

20. STUDY REPORT/PUBLICATIONS 25

21. INTELLECTUAL PROPERTY 25

22. STUDY MILESTONES 27

23. GANTT CHART 28

24. REFERENCES 29

1. RESEARCH PERSONNEL CONTACTS

1.1. Principal Investigator

Name	Dr James Prentis (Consultant Anaesthetist)
Address	Freeman Hospital<br>Newcastle upon Tyne<br>NE77DN
Phone	0191 2448684
Email	james.prentis@nhs.net

1.2. Co-investigators

Name	Dr Gabriel Cucato (exercise physiologist)
Address	Department of Sport, Exercise & Rehabilitation<br>Faculty of Health and Life Sciences<br>Northumbria University<br>Room 259, Northumberland Building<br>Newcastle Upon Tyne<br>NE1 8ST
Phone	01912273371
Email	Gabriel.cucato@northumbria.ac.uk

Name	Dr Chris Snowden (consultant anaesthetist)
Address	Freeman Hospital<br>Newcastle upon Tyne<br>NE77DN
Phone	0191 2336161
Email	chris.snowden1@nhs.net

Name	Ms Emma McCone (pre-operative lead nurse)
Address	Freeman Hospital<br>Newcastle upon Tyne<br>NE77DN
Phone	0191 2336161
Email	emma.mccone@nhs.net

Name	Mr Craig Nesbitt (consultant vascular surgeon)
Address	Freeman Hospital<br>Newcastle upon Tyne<br>NE77DN
Phone	0191 233 6161
Email	craig.nesbitt@nhs.net

Name	Mr Sandip Nandhra (NIHR Fellow – vascular surgery)
Address	Freeman Hospital<br>Newcastle upon Tyne<br>NE7 7DN
Phone	0191 233 6161
Email	Sandip.nandhra@nhs.net

Name	Prof John Saxton (Professor of Clinical Exercise Physiology)
Address	Department of Sport, Exercise & Rehabilitation<br>Faculty of Health and Life Sciences<br>Northumbria University<br>Room 259, Northumberland Building<br>Newcastle Upon Tyne<br>NE1 8ST
Phone	01912273371
Email	john.saxton@northumbria.ac.uk

Name	Mr Paul Court (chief executive)
Address	Healthworks NE<br>The Health Resource Centre<br>Adelaide Terrace<br>Newcastle upon Tyne<br>NE4 8BE
Phone	0191 272 4244
Email	paul.court@hwn.org.uk

Name	Mr Oliver Bell (Acting Health and Wellbeing Manager)
Address	Newcastle United Foundation<br>St. James's Park<br>Newcastle upon Tyne<br>NE1 4ST
Email	oliver.bell@nufc.co.uk

Name	Dr Mackenzie Fong (research fellow, NIHR Applied Research Collaboration North East and North Cumbria)
Address	Population Health Sciences Institute<br>Faculty of Medical Sciences<br>Newcastle University<br>William Leech Building<br>Newcastle upon Tyne<br>NE2 4HH
Email	mackenzie.fong@newcastle.ac.uk

Name	Professor Eileen Kaner (Professor of public health and primary care research)
Address	Population Health Sciences Institute<br>Faculty of Medical Sciences<br>Newcastle University<br>Baddiley-Clark Building<br>Newcastle upon Tyne<br>NE2 4AX
Email	eileen.kaner@newcastle.ac.uk

Name	Maisie Rowland (research assistant)
Address	Population Health Sciences Institute<br>Faculty of Medical Sciences<br>Newcastle University<br>William Leech Building<br>Newcastle upon Tyne<br>NE2 4HH
Email	Maisie.Rowland@newcastle.ac.uk

Name	Dr Nawaraj Bhattarai (research fellow, NIHR Applied Research Collaboration North East and North Cumbria)
Address	Population Health Sciences Institute<br>Faculty of Medical Sciences<br>Newcastle University<br>Baddiley-Clark Building<br>Newcastle upon Tyne<br>NE2 4AX
Email	nawaraj.bhattarai@newcastle.ac.uk

2. PROTOCOL SIGNATURE PAGE

2.1. Principal investigator

Name……………………………………Signature…………………………… Date…………

I confirm that I have read and understood protocol version 1.6 dated 19/03/2021. I agree to comply with the study protocol, principles of Good Clinical Practice (GCP), research governance, clinical trials regulations and appropriate reporting requirements

2.2. Co-investigators

Name……………………………………Signature…………………………… Date…………
Name……………………………………Signature…………………………… Date…………
Name……………………………………Signature…………………………… Date…………
Name……………………………………Signature…………………………… Date…………
Name……………………………………Signature…………………………… Date…………
Name……………………………………Signature…………………………… Date…………
Name……………………………………Signature…………………………… Date…………
Name……………………………………Signature…………………………… Date…………
Name……………………………………Signature…………………………… Date…………
Name……………………………………Signature…………………………… Date…………

3. BRIEF PROTOCOL SUMMARY

**Short Title**	TEXT-PAD study
**Protocol Version**	Version 1.6
**Protocol Date**	19/03/2021
**Funding Source**	League of friends of freeman hospital and departmental funding
**Study design**	Feasibility study and pilot randomised control trial with embedded process evaluation
**Study Intervention**	A 12-week home-based telehealth behavioural intervention delivered in low SES patients with PAD
**Primary objectives**	To assess the feasibility and acceptability of a 12-week home-based telehealth behavioural intervention delivered in low SES patients with PAD
**Secondary objectives**	1. To determine the preliminary effectiveness of a 12-week home-based telehealth behavioural intervention compared to usual care, including effect on: functional status, tobacco and alcohol use, quality of life, dietary quality, mental wellbeing and daily ambulatory physical activity levels<br>2. To develop and test tools to measure the costs and effects for the health economic evaluation of 12-week telehealth behavioural intervention compared to usual care.
**Study site**	Single centre study – Newcastle upon Tyne NHS trust
**Study population**	Patients attending the vascular outpatient clinic at Freeman Hospital with a confirmed diagnosis of PAD and from lowest 30% of super-output areas
**Study duration**	12 months
**Sponsor**	Newcastle upon Tyne NHS Trust

4. BACKGROUND

Peripheral arterial disease (PAD) results from chronic atherosclerosis that progressively leads to partial or total obstruction of the arteries, thereby, reducing blood flow and oxygen delivery to the peripheral regions of the body (1). The main symptom of PAD is intermittent claudication, characterized by pain, cramp, or burning that occurs in the lower limbs during walking exercise and is relieved by rest (2). Due to these symptoms, patients with PAD experience lower physical function (3), impaired cardiovascular function and lower quality of life (4). Based on evidence demonstrating the benefits of exercise on walking capacity (5), cardiovascular function (6, 7) and quality of life (5), supervised exercise training is recommended by the National Institute for Health and Clinical Excellence (NICE) as first line treatment of claudication caused by PAD (8). NICE also recommends that patients with PAD are offered advice, support, and treatment for the secondary prevention of cardiovascular disease (CVD), including smoking cessation, diet, weight management, and exercise (8). Despite these recommendations, supervised exercise is rarely delivered in the clinical setting and, patients are not systematically offered evidence-based interventions that support behavioural modification. Ineffectual provision of and, referral to, behaviour change programmes may contribute to further deterioration of health, leading many patients to undergo surgical procedures for PAD management e.g. stent or bypass. While these procedures may provide short term benefits, they incur greater risk and cost (9, 10). Also, without comprehensive behavioural intervention, patients are likely to resume the same poor health behaviours that contributed to PAD development initially. Therefore, there is an imperative to develop, evaluate and integrate into care pathways 'prehabilitation' interventions that optimise patient health, prevent further deterioration and improve long-term outcomes. Given the systematic relationship between deprivation and health, these programmes are accessible and acceptable to socioeconomically deprived patients.

Usually, supervised exercise programmes and behavioural interventions are delivered face-to-face. However, the current covid-19 pandemic has significantly changed the organizational structure of health institutions and diverted attention to pandemic management (11). There has also been reports of outbreaks during exercise sessions, limiting face-to-face delivery of care to high-risk populations until a vaccine has been developed (12). Telephone-health (tele-health) enables patients to access care remotely via digital platforms. It represents a 'COVID-proof', cost-effective and scalable care delivery option (13) and mitigates some traditional barriers to access e.g. living remotely.

This pilot RCT will establish the feasibility and acceptability of a 12-week home-based telehealth exercise and behavioural intervention delivered in low SES patients with PAD. The programme will be developed and delivered collaboratively between Newcastle upon Tyne NHS Trust, Northumbria University, Newcastle University, Healthworks and Newcastle United Foundation Club (NUFC) Foundation. The use of premier football team branding has been shown to improve the effectiveness of exercise and weight loss interventions (12,13) and improve recruitment of 'hard to engage' men (14). This study will also determine the preliminary effectiveness of the intervention for improving clinical and health outcomes.

5. PRIMARY RESEARCH QUESTION

Is it feasible to deliver a 12-week homebased multimodal telehealth behavioural intervention in low SES patients with PAD and, is the intervention and study acceptable to patients?

6. AIMS and OBJECTIVES OF THE STUDY

6.1. Primary aim

To investigate the feasibility and acceptability of a 12-week homebased multimodal telehealth behavioural intervention in low SES patients with PAD.

6.2. Primary objectives

To determine:

- Rates of patient screening, eligibility, recruitment, and retention to 12-week follow-up
- Patient compliance to the intervention (number of sessions attended and completed)
- Patient acceptability of the intervention through semi-structured qualitative interviews

6.3. Secondary aims

To determine the preliminary effectiveness of a 12-week homebased telehealth behavioural intervention compared to usual care.

6.4. Secondary objectives

To investigate whether a 12-week homebased multimodal telehealth behavioural intervention compared to usual care:

- Improves functional capacity
- Reduces alcohol and tobacco use
- Improve diet quality
- Improves quality of life and mental wellbeing
- Increases daily ambulatory physical activity levels

6.5. Tertiary aim

To explore the measurement of resource utilisation, costs and effects in an economic evaluation that would be conducted as part of a definitive RCT.

6.6. Tertiary objectives

To develop and test tools to measure the costs and effects for the health economic evaluation of 12-week telehealth behavioural intervention compared to usual care.

7. STUDY DESIGN

This is a single centred feasibility study and pilot randomised control trial assessing a 12-week telehealth behavioural intervention delivered in low SES patients with PAD. A logic model for this project is shown in Figure 1.

7.1. Participants

Patient eligibility will be based on the criteria below.

Inclusion criteria

- Diagnosis of PAD confirmed by ankle brachial index <0.90 in one or both limbs
- Age >= 40 years
- Able to walk distance >50m
- Live in an area deemed in lowest 30% of super output area from Office of National Statistics

Exclusion criteria

- chronic limb threatening ischemia
- short claudication distance <50m
- severe heart disease (Grade III or IV, New York Heart Association)

- severe ischemic or haemorrhagic stroke or neurodegenerative diseases
- severe hypertension (systolic blood pressure of more than 180 mm Hg, and a diastolic blood pressure of more than 100 mm Hg)
- uncontrolled cardiac arrhythmias (unstable angina during the previous month and myocardial infarction during the previous month)
- a resting heart rate of more than 120 beats per minute
- has already undergone angioplasty, bypass or other surgical intervention for PAD
- other severe comorbid conditions preventing the ability to engage in physical activity, inability or unwillingness to undertake the commitments of the study

7.2. Research staff

The core research team based within the Newcastle upon Tyne NHS Trust or Northumbria University.

- Dr James Prentis
- Dr Gabriel Cacuto
- Dr Chris Snowden
- Mr Craig Nesbitt
- Mr Sandip Nandha
- Ms Emma McCone
- Prof John Saxton
- Mr Paul Court
- Mr Oliver Bell
- Dr Mackenzie Fong
- Professor Eileen Kaner
- Dr Nawaraj Bhattarai
- Ms Maisie Rowland

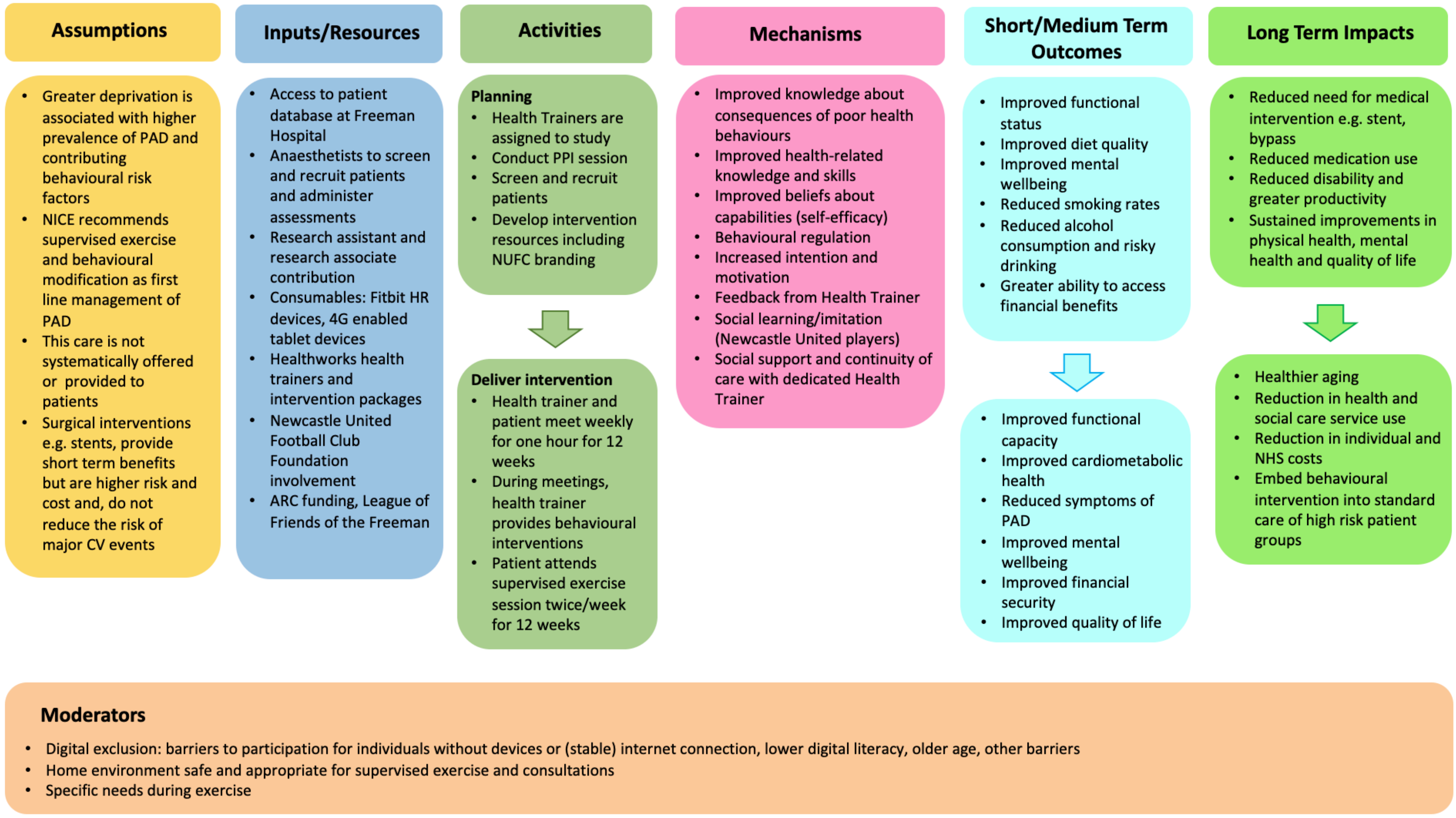

Figure 1. A logic model of pilot RCT investigating the feasibility, acceptability and preliminary effectiveness of a 12-week multimodal telehealth behavioural intervention in low SES patients with PAD.

7.3. Recruitment procedures

A comprehensive list of patients with PAD attending the Freeman Hospital is already kept. The research team will assess potential suitability and contact the participants by telephone. If the participant expresses interest in the study the PIS and consent form will be forwarded on. Hospital visits will be undertaken for formal consent (baseline) and the follow-up outcome assessments.

7.4. Consent

Patients will be provided with appropriate participant information sheets designed in compliance with national guidance. They will have adequate time to consider the information, ask questions and have them answered sufficiently. Patients will be advised that participation is voluntary and that they may withdraw from the study at any time without having to provide a reason or affecting their care. Patients who are willing to participate will be asked to sign a consent form, and this process will conducted by a trained delegated member of the research team. A copy of the signed consent form and participant information sheet will be filed in the patients' medical notes and a further copy will be given to the patient. The original signed consent form will be retained in the Investigator site file and the patients' GP will be informed of their participation in the study.

7.5. Randomisation

Patients will be randomised in a 1:1 ratio to either the telehealth multimodal lifestyle intervention or standard care. The randomization will be performed in blocks of 15 patients, using an online randomisation generator (*www.randomizer.org*).

8. INTERVENTION

The intervention has been co-designed with Newcastle upon Tyne NHS Trust, Northumbria University, Healthworks NE and the Newcastle United Foundation. Patients in the intervention group will receive educational materials and videos outlining the main intervention components and how health behaviours impact upon their condition. The videos will be NUFC/Healthworks branded and be developed by the individuals from these organizations. Patients will also receive a NUFC foundation T-shirt. Shortly after allocation to the intervention group, patients will be contacted by a Health Trainer from Healthworks who will

conduct an initial assessment and consultation. Health trainers have various qualifications in health care e.g. nutrition degree, Level 4 rehabilitation qualification, all receive Healthworks training in motivational interviewing and intervention delivery. Patients will meet with their dedicated health trainer weekly for one hour via phone call/videoconference for 12 weeks and discuss the behaviours outlined below. Health trainers use many behaviour change techniques to promote modification of risk factors such as goal setting, problem solving and self-regulation.

Smoking Cessation

Self-reported smoking habits will be assessed at baseline. Patients who smoke will receive a cessation intervention from the health trainer i.e. discussion of previous quit attempts and benefits of quitting to aid in improving health and exercise capacity. If required, nicotine replacement therapy vouchers which are redeemable at their local pharmacy will be posted to the patient. An eight-week supply of e-cigarette cartridges may also supplied.

Alcohol intervention

Health trainers will deliver a previously evaluated brief behavioural intervention to reduce alcohol intake to low-risk levels (<14 units per week) (15). Intervention materials incorporate specific techniques that target intention formation and enactment of behaviour change (e.g. information on health consequences, social support, goal setting behaviour, problem solving, restructuring the physical environment). Patients suspected to have an alcohol use disorder or risky drinking at baseline will receive additional intervention from their GP.

Nutrition

Patients will receive basic nutrition education and health eating advice in line with recommendations from the British Heart Foundation and Diabetes UK. Health Trainers will provide help to overcome barriers to healthy eating.

Mental well-being and finances

Patients will receive a light-touch intervention on sleep hygiene and stress management. If we discover that the patient is severely depressed, has self-harming concerns or suicidal thoughts the GP will be contacted, or patient signposted for further help. Health trainers will also help

patients to determine their eligibility to receive benefits and, facilitate access and uptake if required.

Supervised exercise

The home-based exercise training will be performed twice a week for 12 weeks via Zoom (up to 5 patients per session). Each session will be comprised of warm-up (10 min), the main part (15 to 20 min), and cooldown (5 to 10 min). The training aim to develop resistance, aerobic and functional capacity such as getting up, walking, pulling, pushing, throwing, transferring body weight or external loads. An example of multimodal exercise training is shown in Box 1.

The training intensity will be progressively adjusted by increasing the load (e.g. using common household objects), increasing the complexity, speed of movements and volume of exercises by varying circuits. The intensity of the exercise will be monitored using the Borg scale (from 0 to 20) with target intensity zone from 12 to 14 (Somewhat hard to hard) (16).

In addition to the home-based training sessions, patients will be encouraged to increase their physical activity. Patients will be provided with a Fitbit device to monitor their step count and will be recommended to increase their previous week's average step count by 10%. Participants who have access to the internet will be asked to upload data to the Fitabase research platform. Data will be anonymised and will only be accessible to the research number.

8.1. Usual care

Patients randomized to the standard care group will receive general recommendations (**Box 2**) to modify risk factor and standard care as per trust guidelines given in their routine outpatient clinic appointment. Patients will also receive specific advice to perform unsupervised walking exercise for around 30 minutes three to five times a week, according to recently published NICE guidelines (8). Patients will also receive a Fitbit device so they can measure their own exercise capacity and increase as recommended. They will be asked to upload their data to the research platform if possible, as per the lifestyle intervention group.

Box 1. Example of exercise training module in the intervention.

Duration	Category	Exercise	Intensity (Borg Scale)
10 min	Warm-up	• Active and dynamic joint mobility; coordination, balance, displacement, spatial orientation and proprioception exercises.	Very light to fairly light
15 to 20 min	Resistance	• Resistance exercise for upper and lower limbs<br>• 6 to 8 exercises<br>• 2-3 sets of 8 to 10 repetitions<br>• Interval sets 1.5-2 min	Somewhat hard to hard
	Flexibility	• Emphasis on joint mobility exercises<br>• Maintenance of static positions combined with breathing techniques;<br>• Proprioceptive neural facilitation techniques<br>• 40 sec to 1 min each exercise	Somewhat hard to hard
	Aerobic exercise (circuit training)	• Global exercises, involving large muscle groups focused on aerobic capacity<br>• Circuit of 3 to 4 exercises<br>• Stimulus – 30 sec to 1 min<br>• Passive interval (1 min)	Somewhat hard to hard
5 to 10 min	Cooldown	• Active and static stretching exercises<br>• Breathing relaxation exercises	Very light to fairly light

Box 2. Walking intervention for patients randomised to standard care.

**Step 1:** Warm up. Stretch your calf and thigh muscles in each leg for 10 to 15 seconds.<br>**Step 2:** Start walking. Walk at a fast-enough pace for about 5 minutes, even though it may cause some mild pain.<br>**Step 3:** Stop and rest. After 5 minutes of mild or moderate pain, stop and rest until the pain goes away.<br>**Step 4:** Repeat the walk-and-stop routine several times. During the first two months of your walking program, build up slowly to walking a total of 35 minutes each session, not counting the rest breaks. Keep adding a few minutes until you're at the goal of walking 50 minutes.<br>**Step 5:** Cool down. Finish by walking slowly for 5 minutes. Then, stretch your calf and thigh muscles again.<br>**Step 6:** Stick with it.

9. OUTCOME MEASUREMENTS

9.1. Primary outcomes

Feasibility will be determined by calculating the rate of patient screening, eligibility, recruitment, retention at 12 weeks and adherence to the intervention (number of sessions attended and completed).

Patient acceptability of the intervention and study experience more broadly will be determined through semi-structured qualitative 1-2-1 interviews and/or focus groups. Given the challenges of conducting focus groups remotely, and anticipated characteristics of the participant group, we expect that 1-2-1 interviews will be more practical and facilitative. We will purposively sample participants from both study arms so that the experiences of patients from varied ethnic backgrounds, age and gender are represented. Acceptability of the intervention will be guided by Sekhon et al.'s Theoretical Framework of Acceptability (17) and the NIH Behavior Change Consortium's Best Practices and Recommendations. We will also ask patients about their experience of participating in the study e.g. informed consent process, time commitment, acceptability of measures e.g. readability, burden etc. We will also ask for their experiences, thoughts and attitudes towards current usual care for PAD. Interviews will be conducted until data saturation is reached in a maximum of 20 participants.

All focus groups will be audio recorded and transcribed, and these data will be analysed thematically to generate themes and outcomes. Participants will receive a £20 voucher as reimbursement for their participation.

9.2. Secondary outcomes

Objective functional capacity

Patients will complete the 6-minute walk test (6MWT) (18) at baseline and 12-week follow-up. Briefly, patients will be encouraged to “walk at their usual pace for six-minutes and cover as much ground as possible” and rest if necessary. The outcomes will be the onset claudication distance (distance walked when the patients related the occurrence of symptom of intermittent claudication (19)) and six-minute total walking distance (6MWD; the maximum distance achieved by the patient at the end of the test). The test will be administered by a trained member of staff and conducted in line with the American Thoracic Society guidance (18). The 6MWT assesses the submaximal level of functional capacity and has good test-retest reliability (19-21), responsiveness (22, 23) and validity (19, 24, 25) in a range of populations including adults with PAD (19).

Subjective functional capacity

Patients will complete the Walking Impairment Questionnaire (WIQ) (26) at baseline and 12-week follow-up to assess three factors of walking impairment: walking distance, walking speed, and the ability to climb stairs. Patients will be asked how difficult it was to walk in these situations should answer as “none, slight, some, much or unable”. Each domain is anchored from 0, representing extreme limitation, to 100 representing no difficulties. The WIQ has been validated for use in patients with PAD (27, 28).

Patients will also complete the Walking Estimated Limitation Calculated by History (WELCH) questionnaire (29) at both time points. Patients report how long they are able to walk at certain speeds, and then how they would rate their speed of walking relative to their relatives, friends or people at same age. The WELCH has been validated for use in patients with intermittent claudication (30).

Quality of life

Patients will complete the vascular quality of life questionnaire (VascuQoL-6) (31) at baseline and 12-week follow-up. The measure is composed of six items evaluating the impact of vascular disease on social aspects and capacity to perform daily activities. Each item is scored 1-4. The total score is achieved by summarizing the score on each item, resulting in a score between 6 and 24. Higher value indicates better health status. The instrument has good test-retest reliability and validity in patients with PAD (32, 33).

Patients will also complete the EuroQoL questionnaire (EQ-5D-5L) (34) at both study time points which measures five dimensions: mobility, self-care, usual activities, pain/discomfort and anxiety/depression. The digits for the five dimensions can be combined into a 5-digit number that describes the patient's health state (35). The EQ-5D-5L has been widely validated (34, 36) including in patients with cardiovascular disease (37) and as it is used extensively in health research, its inclusion in the current study will enable comparison with previous literature.

Dietary quality

Participants will complete the Short Form Dietary Questionnaire (38) which comprises 24 items that collect data on dietary intake frequency. Participants' responses will be used to derive a dietary quality score. The tool is shown to be a valid method of assessing dietary quality in UK adults (38).

Physical activity levels

Daily ambulatory activity will be assessed using a wrist-worn accelerometer (Fitbit Charge HR) which measures step count, resting heart rate and time in sedentary behaviour as well as light, moderate and vigorous activities. Fitbit devices have been shown to have good concurrent validity for measuring sedentary behaviour and physical activity compared to research-grade accelerometers (39-41). Patients will be instructed to wear the device every day during the study and asked to charge the device every 48-36 hours overnight. Patients' anonymised activity data will be uploaded to the Fitbit online dashboard (Fitabase) and extracted by researchers for analyses.

Sleep

Sleep quality and quantity will also be recorded by the Fitbit device. Patients' anonymised sleep data will be uploaded to Fitabase and extracted by researchers for analyses. Wrist-worn Fitbit devices are shown to have good validity for obtaining gross estimates of sleep parameters (41, 42).

Alcohol and tobacco use

The 3-item Alcohol Use Disorders Identification Test—Consumption (AUDIT-C) screening tool (43) will be administered to patients at baseline and 12-week follow-up to identify alcohol use disorders or risky drinking. The tool has good validity in diverse populations (44, 45). If the patient is suspected to have an alcohol use disorder or risky drinking based on their AUDIT-C score, their general practitioner will be contacted to offer further input and advice.

Patients will complete a standard 30 second carbon monoxide breath test at baseline and follow- up to formally assess smoking status. This is not an aerosol generating procedure and therefore will be appropriate to use.

Self-reported smoking habits will be assessed at baseline and follow-up. Patients will be asked to report their current tobacco smoking status and frequency of smoking cigarettes/other tobacco products and, previous tobacco smoking status.

Mental wellbeing

Patients will complete the 14-item hospital anxiety-depression score (HADS) (46) at baseline and 12-week follow-up. The 14-item instrument is comprised of a depression subscale and anxiety subscale which are each assessed through 7-items. Respondents are asked to rate their mental and emotional state over the past week. The HADS has good validity in community and clinical populations (47).

Patient activation

The Patient Activation Measure (PAM®) (48) measures patients' knowledge, skills and confidence in managing their condition. PAM licences are available from NHS England and Improvement as part of the Supported Self-management component of the Personalised Care

Programme. At baseline and 12-week follow-up patients will respond (strongly disagree/disagree/agree/strongly agree/N/A) to 13 statements related to their confidence in managing their health. Patients' PAM score (0 - 100) will place them within one of four activation categories, providing insight into a range of health-related characteristics and behaviours. The PAM has been validated for use in adults with long term conditions (49-51).

Resource utilisation

The case report forms (CRF) will measure the resource utilisations by each patient. The CRF will be administered to each patient at baseline and 12-week follow-up in both arms of the trial asking them to report any health care resources they have utilised in the preceding 12 weeks. Unit costs from secondary sources will be applied to the resources utilised to generate costs incurred by each patient.

10. STUDY POWER

As per good practice recommendations for pilot studies (52), we will aim to recruit 30 patients to each arm and obtain a total sample of 60 participants. Findings of the current study will be used to inform the power calculations of future definitive RCTs.

11. DATA ANALYSIS

Primary outcome data will be reported as descriptive statistics, including rates of: patient screening, eligibility, recruitment and retention to 12-week follow-up and survey completion. Qualitative interview data will be analysed thematically to generate themes. Exploratory between-group analyses will be conducted to determine preliminary intervention effectiveness. Normality and homogeneity of variance will be performed using the Shapiro-Wilks and Levene tests, respectively. For comparison of the variables at the pre-intervention, we will perform a one-way ANOVA. To analyze the responses before and after the intervention period, two-way analysis of variance for repeated measures will be used, with the main factors being the group (and the time (pre and post intervention) with Newman-Keuls post-hoc test. The level of significance will be set at $P < 0.05$.

12. HEALTH ECONOMICS

The trial will not be adequately powered to conduct a formal health economic evaluation, therefore the data accrued from the pilot trial will be reported using descriptive statistics to explore the differences in resource utilisations, costs and effects between the trial arms. We will also assess the completion rates for the health economics data collection tools, responses for each question and health state utility values. At baseline and 12-week follow-up, all patients will complete the following:

- EuroQol's EQ-5D-5L and EQ-5D VAS questionnaire
- Case report forms measuring health resource utilisation

13. STUDY MONITORING

- This is a low risk study and major safety issues are not anticipated.
- The study may be subject to inspection and audit by the study sponsor (NUTH).
- This is to ensure that the study is conducted to a high standard in accordance to the protocol, the principles of GCP, relevant regulations, guidelines and with regard to patient safety.

14. SERIOUS ADVERSE EVENT MONITORING AND REPORTING

- RECs require all serious adverse events to be reported. We do not anticipate any major risks or adverse events from this study.
- Should they occur they will be recorded during visits and categorised as to relatedness and severity
- The NUTH NHS Trust will be informed according to trust policy

15. ETHICS AND REGULATORY ISSUES

- Ethical approval from a local REC and R&D bodies will be obtained prior to the commencement of the study.
- Written confirmation of approval will be received before the study commences
- Participant Information Sheets (PIS) will be provided to eligible participants (clinicians and patients) and written informed consent will be obtained prior to any study procedures being undertaken.

16. PATIENT AND PUBLIC INVOLVEMENT

With the time constraints around COVID19 and the issues concerning group-based discussions, PPI groups have been difficult to undertake. However, we have performed a small PPI group among patients with PAD to determine the face-acceptability and value of the intervention.

17. CONFIDENTIALITY & DATA HANDLING

- Personal data will be regarded as strictly confidential
- Identifiable data e.g. name, date of birth and consent form, will be stored separately to study data
- An NHS computer will be used for all inputting of data onto a database. This will be password protected and have IT security measures offered by the Freeman Hospital IT department.
- To preserve anonymity, any data leaving the study site will identify participants by a unique study identification code
- The study will comply with the Data Protection Act 1998 and Caldicott Principles
- All study records and investigator Site Files will be kept at site in a locked filing cabinet with restricted access

18. INFORMATION GOVERNANCE/DIGITAL PLATFORM

- All patients will be consented to transfer data via nhs.net email between NUTH and Healthworks as this is necessary for the running of the study.
- Data will be stored on the REDCAP system
- The data transferred via the fitbits will only contain the patients' unique study identification code and no other patient identifiable data.
- Fitabase resides on Microsoft Azure platform. It relies on robust security, both physical on-premise guarding and over network provided as part of the platform. Windows Azure runs in data centres managed and operated by Microsoft Global Foundation Services (GFS). These geographically dispersed data centres comply with key industry standards such as ISO/IEC 27001:2005 for security and reliability. They are managed, monitored and administered by Microsoft operations staff that have years of experience in delivering the world's largest online services with 24:7 continuity. In addition to data centre, network and personnel security practices, Windows Azure incorporates security practices at the

application and platform layers to enhance security for service administrators. In addition to primary copies of the databases, Small Steps Labs LLC maintains snapshot archives of database for disaster recovery purposes. Backup copies reside only on hardware and are encrypted and password protected.

19. INSURANCE AND FINANCE

- The Newcastle upon Tyne Hospitals NHS Trust has liability for clinical negligence that harms individuals toward whom they have a duty of care
- NHS Indemnity covers NHS staff conducting the trial for potential liability in respect of harm arising from the conduct of the study.
- Grant from League of Friends of Freeman Hospital and departmental resources are funding the project

20. STUDY REPORT/PUBLICATIONS

- Publication will be the responsibility of the PI and co-applicants
- Authorship principles will follow the International Medical Editors conventions as follows: Each author should have participated significantly in the work to take responsibility for the content. This participation should include (a) conception or design, or analysis and interpretation of data, or both; (b) drafting the article or revising it for critically important content; and (c) final approval of the version to be published.
- The outcomes of this study will be published in peer review journals and presented at local, national and international meetings and conferences
- Individuals will not be identified from any study report
- A lay summary of the results will be available for all study participants

21. INTELLECTUAL PROPERTY

This study will generate work that will have intellectual property. Any materials developed will be copyrighted as per Trust property. We will not sell any materials for profit however we will require any user to acknowledge the source of materials.

22. STUDY MILESTONES

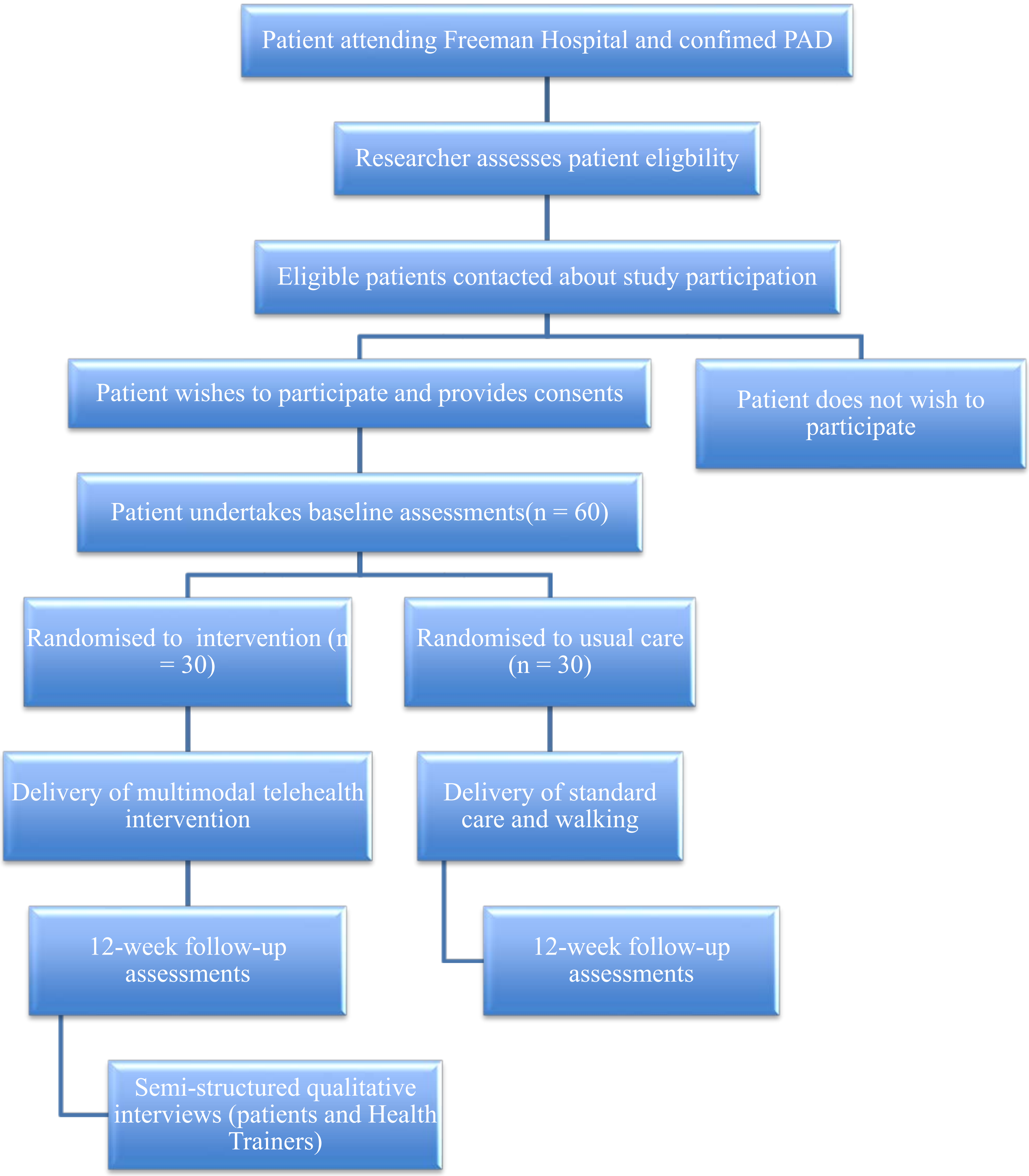

23. <u>GANTT CHART</u>

	2020							2021											
	Jun	Jul	Aug	Sep	Oct	Nov	Dec	Jan	Feb	Mar	Apr	May	Jun	Jul	Aug	Sep	Oct	Nov	Dec
Protocol development	■	■	■	■	■														
Sponsorship application			■																
Ethics application			■																
PIS and consent form development					■	■	■												
Sponsorship confirmation					■														
Ethics approval					■														
ARC funding application					■	■													
Intervention materials development					■	■	■												
Research nurse hired						■													
Purchasing of Fitbits and tablets						■	■												
Health Trainers assigned to study						■	■												
Patient screening								■	■	■	■	■							
Patient recruitment								■	■	■	■	■							
Baseline assessments								■	■	■	■	■							
Intervention/usual care								■	■	■	■	■	■	■	■				
Follow-up assessments											■	■	■	■	■				
Quantitative data cleaning															■	■			
Quantitative data analysis															■	■			
Interviews															■	■			
Interview data transcription															■	■	■		
Thematic analysis of qualitative data																■	■		
Dissemination events																	■	■	■
Report writing																	■	■	■

24. REFERENCES

1. Bradberry JC. Peripheral arterial disease: pathophysiology, risk factors, and role of antithrombotic therapy. J Am Pharm Assoc (2003). 2004;44(2 Suppl 1):S37-44; quiz S-5.
2. Meijer WT, Hoes AW, Rutgers D, Bots ML, Hofman A, Grobbee DE. Peripheral arterial disease in the elderly: The Rotterdam Study. Arteriosclerosis, thrombosis, and vascular biology. 1998;18(2):185-92.
3. Gardner AW, Clancy RJ. The relationship between ankle-brachial index and leisure-time physical activity in patients with intermittent claudication. Angiology. 2006;57(5):539-45.
4. Spronk S, White JV, Bosch JL, Hunink MG. Impact of claudication and its treatment on quality of life. Semin Vasc Surg. 2007;20(1):3-9.
5. Lane R, Harwood A, Watson L, Leng GC. Exercise for intermittent claudication. Cochrane Database Syst Rev. 2017;12:CD000990.
6. Cornelis N, Nassen J, Buys R, Fourneau I, Cornelissen V. The Impact of Supervised Exercise Training on Traditional Cardiovascular Risk Factors in Patients With Intermittent Claudication: A Systematic Review and Meta-Analysis. Eur J Vasc Endovasc Surg. 2019;58(1):75-87.
7. Ritti-Dias RM, Correia MA, Andrade-Lima A, Cucato GG. Exercise as a therapeutic approach to improve blood pressure in patients with peripheral arterial disease: current literature and future directions. Expert Rev Cardiovasc Ther. 2019;17(1):65-73.
8. Layden J, Michaels J, Bermingham S, Higgins B, Guideline Development G. Diagnosis and management of lower limb peripheral arterial disease: summary of NICE guidance. BMJ. 2012;345:e4947.
9. Reynolds MR, Apruzzese P, Galper BZ, Murphy TP, Hirsch AT, Cutlip DE, et al. Cost-effectiveness of supervised exercise, stenting, and optimal medical care for claudication: results from the Claudication: Exercise Versus Endoluminal Revascularization (CLEVER) trial. Journal of the American Heart Association. 2014;3(6):e001233.
10. Mazari FAK, Khan JA, Carradice D, Samuel N, Gohil R, McCollum PT, et al. Economic analysis of a randomized trial of percutaneous angioplasty, supervised exercise or combined treatment for intermittent claudication due to femoropopliteal arterial disease. British Journal of Surgery. 2013;100(9):1172-9.
11. Luo H, Lie Y, Prinzen FW. Surveillance of COVID-19 in the General Population Using an Online Questionnaire: Report From 18,161 Respondents in China. JMIR Public Health Surveill. 2020;6(2):e18576.
12. Brunier A, Harris M. COVID-19 significantly impacts health services for noncommunicable diseases 2020 [Available from: https://www.who.int/news-room/detail/01-06-2020-covid-19-significantly-impacts-health-services-for-noncommunicable-diseases.
13. Peretti A, Amenta F, Tayebati SK, Nittari G, Mahdi SS. Telerehabilitation: Review of the State-of-the-Art and Areas of Application. JMIR Rehabil Assist Technol. 2017;4(2):e7.
14. Pringle A, Zwolinsky S, McKenna J, Robertson S, Daly-Smith A, White A. Health improvement for men and hard-to-engage-men delivered in English Premier League football clubs. Health Education Research. 2014;29(3):503-20.
15. Snowden C, Lynch E, Avery L, Gerrand C, Gilvarry E, Goudie N, et al. Preoperative Behavioural Intervention versus standard care to Reduce Drinking before elective orthopaedic Surgery (PRE-OP BIRDS): protocol for a multicentre pilot randomised controlled trial. Pilot and Feasibility Studies. 2018;4(1):140.
16. Borg GA. Psychophysical bases of perceived exertion. Med Sci Sports Exerc. 1982;14(5):377-81.
17. Sekhon M, Cartwright M, Francis JJ. Acceptability of healthcare interventions: an overview of reviews and development of a theoretical framework.(Report). BMC Health Services Research. 2017;17(1).
18. National Library of M. ATS statement: guidelines for the six-minute walk test. American journal of respiratory and critical care medicine. 2002;166(1):111-7.

19. Montgomery PS, Gardner AW. The clinical utility of a six-minute walk test in peripheral arterial occlusive disease patients. J Am Geriatr Soc. 1998;46(6):706-11.
20. Eng JJ, Dawson AS, Chu KS. Submaximal exercise in persons with stroke: test-retest reliability and concurrent validity with maximal oxygen consumption. Archives of physical medicine and rehabilitation. 2004;85(1):113-8.
21. Steffen T, Hacker T, Mollinger L. Age- and gender-related test performance in community-dwelling elderly people: Six-minute walk test, Berg Balance Scale, Timed Up & Go Test,a nd gait speeds. Physical Therapy. 2002;82(2):128-37.
22. Perera S, Mody SH, Woodman RC, Studenski SA. Meaningful Change and Responsiveness in Common Physical Performance Measures in Older Adults. Journal of the American Geriatrics Society. 2006;54(5):743-9.
23. Casanova C, Cote CG, Marin JM, de Torres JP, Aguirre-Jaime A, Mendez R, et al. The 6-min walking distance: long-term follow up in patients with COPD. The European respiratory journal. 2007;29(3):535-40.
24. Harada ND, Chiu V, Stewart AL. Mobility-related function in older adults: Assessment with a 6-minute walk test. Archives of physical medicine and rehabilitation. 1999;80(7):837-41.
25. Downham D, Flansbjer U-B, Holmback A, Lexell J, Patten C. Reliability of gait performance tests in men and women with hemiparesis after stroke. Journal of Rehabilitation Medicine. 2005;37(2):75-82.
26. Nicolai SP, Kruidenier LM, Rouwet EV, Graffius K, Prins MH, Teijink JA. The walking impairment questionnaire: an effective tool to assess the effect of treatment in patients with intermittent claudication. J Vasc Surg. 2009;50(1):89-94.
27. Sagar SP, Brown PM, Zelt DT, Pickett WL, Tranmer JE. Further clinical validation of the walking impairment questionnaire for classification of walking performance in patients with peripheral artery disease. Int J Vasc Med. 2012;2012:190641-.
28. Myers SA, Johanning JM, Stergiou N, Lynch TG, Longo GM, Pipinos II. Claudication distances and the Walking Impairment Questionnaire best describe the ambulatory limitations in patients with symptomatic peripheral arterial disease. Journal of vascular surgery. 2008;47(3):550-5.e1.
29. Tew GA, Nawaz S, Humphreys L, Ouedraogo N, Abraham P. Validation of the English version of the Walking Estimated-Limitation Calculated by History (WELCH) questionnaire in patients with intermittent claudication. Vasc Med. 2014;19(1):27-32.
30. Tew GA, Nawaz S, Humphreys L, Ouedraogo N, Abraham P. Validation of the English version of the Walking Estimated-Limitation Calculated by History (WELCH) questionnaire in patients with intermittent claudication. Vascular Medicine. 2014;19(1):27-32.
31. Nordanstig J, Wann-Hansson C, Karlsson J, Lundstrom M, Pettersson M, Morgan MB. Vascular Quality of Life Questionnaire-6 facilitates health-related quality of life assessment in peripheral arterial disease. J Vasc Surg. 2014;59(3):700-7.
32. Kumlien C, Nordanstig J, LundstrAaAaAeA m M, Pettersson M. Validity and test retest reliability of the vascular quality of life Questionnaire-6: a short form of a disease-specific health-related quality of life instrument for patients with peripheral arterial disease.(Report). Health and Quality of Life Outcomes. 2017;15(1).
33. Larsen ASF, Reiersen AT, Jacobsen MB, Kløw N-E, Nordanstig J, Morgan M, et al. Validation of the Vascular quality of life questionnaire - 6 for clinical use in patients with lower limb peripheral arterial disease. Health and quality of life outcomes. 2017;15(1):184.
34. Herdman M, Gudex C, Lloyd A, Janssen M, Kind P, Parkin D, et al. Development and preliminary testing of the new five-level version of EQ-5D (EQ-5D-5L). Quality of Life Research. 2011;20(10):1727-36.
35. van Hout B, Janssen MF, Feng YS, Kohlmann T, Busschbach J, Golicki D, et al. Interim scoring for the EQ-5D-5L: mapping the EQ-5D-5L to EQ-5D-3L value sets. Value Health. 2012;15(5):708-15.
36. Sayah F, Qiu W, Xie F, Johnson J. Comparative performance of the EQ-5D-5L and SF-6D index scores in adults with type 2 diabetes. Quality of Life Research. 2017;26(8):2057-66.

37. Janssen MF, Pickard AS, Golicki D, Gudex C, Niewada M, Scalone L, et al. Measurement properties of the EQ-5D-5L compared to the EQ-5D-3L across eight patient groups: a multi-country study. Quality of life research : an international journal of quality of life aspects of treatment, care and rehabilitation. 2013;22(7):1717-27.

38. Cleghorn CL, Harrison RA, Ransley JK, Wilkinson S, Thomas J, Cade JE. Can a dietary quality score derived from a short-form FFQ assess dietary quality in UK adult population surveys? Public Health Nutrition. 2016;19(16):2915-23.

39. Redenius N, Kim Y, Byun W. Concurrent validity of the Fitbit for assessing sedentary behavior and moderate-to-vigorous physical activity. BMC Medical Research Methodology. 2019;19(1):29.

40. Brewer W, Swanson BT, Ortiz A. Validity of Fitbit’s active minutes as compared with a research-grade accelerometer and self-reported measures. BMJ Open Sport & Exercise Medicine. 2017;3(1):e000254.

41. Tedesco S, Sica M, Ancillao A, Timmons S, Barton J, O'Flynn B. Validity Evaluation of the Fitbit Charge2 and the Garmin vivosmart HR+ in Free-Living Environments in an Older Adult Cohort. JMIR Mhealth Uhealth. 2019;7(6):e13084.

42. Haghayegh S, Khoshnevis S, Smolensky MH, Diller KR, Castriotta RJ. Accuracy of Wristband Fitbit Models in Assessing Sleep: Systematic Review and Meta-Analysis. Journal of medical Internet research. 2019;21(11):e16273-e.

43. Bush K, Kivlahan DR, McDonell MB, Fihn SD, Bradley KA, Project ftACQI. The AUDIT Alcohol Consumption Questions (AUDIT-C): An Effective Brief Screening Test for Problem Drinking. Archives of Internal Medicine. 1998;158(16):1789-95.

44. Bradley KA, Bush KR, Epler AJ, Dobie DJ, Davis TM, Sporleder JL, et al. Two brief alcohol-screening tests From the Alcohol Use Disorders Identification Test (AUDIT): validation in a female Veterans Affairs patient population. Arch Intern Med. 2003;163(7):821-9.

45. Frank D, DeBenedetti AF, Volk RJ, Williams EC, Kivlahan DR, Bradley KA. Effectiveness of the AUDIT-C as a screening test for alcohol misuse in three race/ethnic groups. Journal of general internal medicine. 2008;23(6):781-7.

46. Zigmond AS, Snaith RP. The Hospital Anxiety and Depression Scale. Acta Psychiatrica Scandinavica. 1983;67(6):361-70.

47. Bjelland I, Dahl AA, Haug TT, Neckelmann D. The validity of the Hospital Anxiety and Depression Scale: An updated literature review. Journal of Psychosomatic Research. 2002;52(2):69-77.

48. Hibbard JH, Mahoney ER, Stockard J, Tusler M. Development and testing of a short form of the patient activation measure. Health Serv Res. 2005;40(6 Pt 1):1918-30.

49. Moljord IEO, Lara-Cabrera ML, Perestelo-Pérez L, Rivero-Santana A, Eriksen L, Linaker OM. Psychometric properties of the Patient Activation Measure-13 among out-patients waiting for mental health treatment: A validation study in Norway. Patient Education and Counseling. 2015;98(11):1410-7.

50. Hellström A, Kassaye Tessma M, Flink M, Dahlgren A, Schildmeijer K, Ekstedt M. Validation of the patient activation measure in patients at discharge from hospitals and at distance from hospital care in Sweden. BMC Public Health. 2019;19(1):1701.

51. Ngooi BX, Packer TL, Kephart G, Warner G, Koh KWL, Wong RCC, et al. Validation of the Patient Activation Measure (PAM-13) among adults with cardiac conditions in Singapore. Quality of Life Research. 2017;26(4):1071-80.

52. Lancaster GA, Dodd S, Williamson PR. Design and analysis of pilot studies: recommendations for good practice. J Eval Clin Pract. 2004;10(2):307-12.
