# Supplementary material for: Evaluating the feasibility and acceptability of an exercise and behaviour change intervention in socioeconomically deprived patients with peripheral arterial disease: The textpad study protocol
Source: PLoS One. 2022 Jun 24;17(6):e0269999. doi: 10.1371/journal.pone.0269999 (PMC9231723; doi:10.1371/journal.pone.0269999)
Supplement: Attachment — Submitted filename: Response to Reviewers.docx [file pone.0269999.s003.docx]

Dear Yoshihiro Fukumoto

Please find enclosed the new version of the manuscript: “Evaluating the feasibility and acceptability of an exercise and behaviour change intervention in socioeconomically deprived patients with peripheral arterial disease: THE TEXTPAD STUDY PROTOCOL”.

We have revised the manuscript according to the reviewer’s comments and have included the change within the manuscript which is highlighted in **yellow**. We hope you find this paper acceptable for publication. Thank you for your time and consideration.

Sincerely,

Reviewer #1: This is a protocol paper investigating about the feasibility and acceptability of an exercise and behavior change intervention in patients with peripheral arterial disease (PAD). This research focused on patients with PAD in socioeconomic deprived area. The protocol was arranged well in detail. There were some points to be addressed.

1) Introduction
\# What is the reason for the limitation of study patients to socioeconomically deprived area? More concise explanation about it should be added.

**Answer:** We only included patients in socioeconomically deprived areas because they present poor health status, presence of multiple risk factors, unhealthy lifestyle and more barriers to behaviour change programs [1, 2] when compared to patients living in affluent areas. We wanted to ensure that this type of intervention is feasible and that it promotes preliminary evidence of effectiveness on overall health in deprived areas before we appropriate it for all patients with PAD regardless of socioeconomic status.

2)Method
\# The issue about “uncontrolled cardiac arrhythmias” were hard to understand.
What is the association between “uncontrolled cardiac arrhythmias” and “unstable angina or myocardial infarction”, which was mentioned in parentheses?

**Answer**: We would like to be sorry about this mistake. We correct this error in the new version of the manuscript.

3) #This study uses telehealth devise such as zoom application or fitbit device. How about the preparation of these devices? In addition, patients with low cognitive function might have a hard time handling these devices. Please comment about how to handle the disparity in the availability for IT devices.

**Answer**: On the day that patients are recruited, our team provides all information regarding how to use the smartwatch (Fitbit) and how to access classes via zoom. In addition, HealthWorks offers telephone support for patients experiencing difficulties logging into classes. There have been only a few issues with exercise class access, which our team has quickly resolved.

4) Discussion
\# Authors described the barrier of cardiac rehabilitation in PAD. More concise documentation for the barrier should be discussed. Indeed, there are several levels of the barriers (patient level and physician level) (Expert Rev Cardiovasc Ther. 2020 Nov;18(11):777-789). In addition, covid infection worsened the difficulty in delivering cardiac rehabilitation to each patient.

**Answer:** As recommended, we discussed in more detail the possible barrier to exercise training in PAD patients.

Reviewer #2:

1) In the present manuscript submitted by Cucato et al., there are typos and wrong line spaces a lot, and the reference list is lacking. It looks like a draft before submission. Was the present manuscript reviewed by all authors before submission? I feel that the authors should format the description in whole.

**Answer**: We would like to thank you for your comments. All authors approved the final version of the manuscript. However, we made a new author’s review and updated references and manuscript formatting.

REFERENCES

1.	Foster HME, Celis-Morales CA, Nicholl BI, Petermann-Rocha F, Pell JP, Gill JMR, et al. The effect of socioeconomic deprivation on the association between an extended measurement of unhealthy lifestyle factors and health outcomes: a prospective analysis of the UK Biobank cohort. Lancet Public Health. 2018;3(12):E576-E85. doi: 10.1016/S2468-2667(18)30200-7. PubMed PMID: WOS:000452029600011.

2.	Michie S, Jochelson K, Markham WA, Bridle C. Low-income groups and behaviour change interventions: a review of intervention content, effectiveness and theoretical frameworks. J Epidemiol Commun H. 2009;63(8):610-22. doi: 10.1136/jech.2008.078725. PubMed PMID: WOS:000267941400007.
